# HOW ARE OLDER PEOPLE FARING POST-PANDEMIC? EVIDENCE FROM THE ENGLISH LONGITUDINAL STUDY OF AGING

**DOI:** 10.1093/geroni/igad104.1375

**Published:** 2023-12-21

**Authors:** Giorgio Di Gessa, Paola Zaninotto, Paola Zaninotto

## Abstract

This session provides insights into the experiences of older people in England post-COVID-19 pandemic. Evidence suggests that stay-at-home and lockdown measures during the pandemic impacted several aspects of people’s lives with detrimental consequences for their wellbeing and mental health, particularly among older people. Little is known, however, on whether and to what extent there has been a return to ‘normal life’ since COVID-19 restrictions were lifted. Using data from the English Longitudinal Study of Ageing (ELSA) this symposium aims to provide new evidence on the experiences of older English people since the successful and rapid COVID-19 vaccination rollout and the easing of restrictions in England through the second half of 2021. ELSA is an ongoing longitudinal biennial survey representative of individuals aged 50 and over in private households that has collected data before the pandemic (with the most recent pre-COVID-19 data collected in 2018/19), during the pandemic (on two separate occasions in June/July and November/December 2020), and post-COVID-19 pandemic (with interviews taking place between October 2021 and March 2023). Exploiting the richness of this dataset, this symposium will present the most up-to-date picture of how older people are faring post-pandemic. Results will focus on mental health and wellbeing, social isolation and loneliness, employment and financial status, as well as social participation (including volunteering, caring, and grandparental childcare).

